# Polymeric potassium triformatocobalt(II)

**DOI:** 10.1107/S1600536811008737

**Published:** 2011-03-12

**Authors:** Susanne Wöhlert, Mario Wriedt, Inke Jess, Christian Näther

**Affiliations:** a Institut für Anorganische Chemie, Christian-Albrechts-Universität Kiel, Max-Eyth-Strasse 2, 24118 Kiel, Germany; b Departement of Chemistry, Texas A&M University, College Station, Texas 77843, USA

## Abstract

In the crystal structure of the title compound, poly[tri-μ-formato-cobalt(II)potassium], [CoK(CHO_2_)_3_]_*n*_ the Co^2+^ cations are coordinated by six O-bonded formate anions in an octa­hedral coordination mode and the K^+^ cations are eightfold coordinated by seven O-bonded formate anions within irregular polyhedra. The Co^2+^ cations are connected by bridging formate anions into a three-dimensional coordination network in which the K^+^ cations are embedded. The asymmetric unit consits of one Co^2+^ cation located on a center of inversion, one K^+^ cation located on a twofold axis and two crystallographically independent formato anions, of which one is located on a twofold axis and the other occupies a general position.

## Related literature

For background to this work see: Boeckmann *et al.* (2010) ▶; Wriedt & Näther (2010 ▶); Wriedt *et al.* (2009) ▶. For structures of bimetallic compounds based on potassium formate, see: Antsyshkina *et al.* (1983 ▶); Leontiev *et al.* (1988 ▶). For a description of the Cambridge Structural Database, see: Allen (2002 ▶).
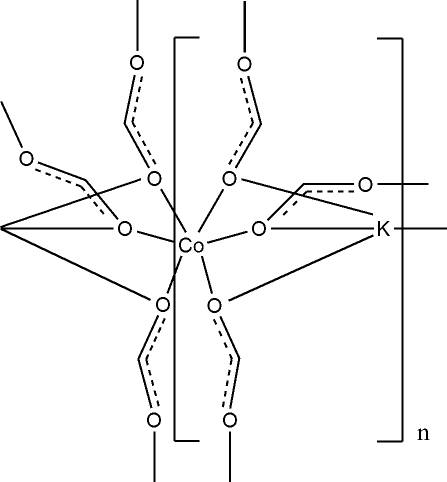


## Experimental

### 

#### Crystal data

[CoK(CHO_2_)_3_]
*M* *_r_* = 233.08
Monoclinic, 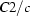

*a* = 10.7244 (8) Å
*b* = 8.9653 (6) Å
*c* = 6.8742 (5) Å
β = 95.539 (6)°
*V* = 657.85 (8) Å^3^
*Z* = 4
Mo *K*α radiation
μ = 3.22 mm^−1^
*T* = 293 K
0.16 × 0.09 × 0.06 mm

#### Data collection

Stoe IPDS-2 diffractometer
Absorption correction: numerical (*X-SHAPE* and *X-RED32*; Stoe & Cie, 2008) ▶ *T*_min_ = 0.711, *T*_max_ = 0.817
6120 measured reflections
892 independent reflections
853 reflections with *I* > 2σ(*I*)
*R*_int_ = 0.031

#### Refinement

*R*[*F* ^2^ > 2σ(*F* ^2^)] = 0.020
*wR*(*F* ^2^) = 0.046
*S* = 1.15
892 reflections
54 parameters
H-atom parameters constrained
Δρ_max_ = 0.25 e Å^−3^
Δρ_min_ = −0.57 e Å^−3^

### 

Data collection: *X-AREA* (Stoe & Cie, 2008) ▶; cell refinement: *X-AREA* ▶; data reduction: *X-AREA* ▶; program(s) used to solve structure: *SHELXS97* (Sheldrick, 2008 ▶); program(s) used to refine structure: *SHELXL97* (Sheldrick, 2008 ▶); molecular graphics: *XP* in *SHELXTL* (Sheldrick, 2008 ▶) and *DIAMOND* (Brandenburg, 1999 ▶); software used to prepare material for publication: *XCIF* in *SHELXTL*.

## Supplementary Material

Crystal structure: contains datablocks I, global. DOI: 10.1107/S1600536811008737/kj2172sup1.cif

Structure factors: contains datablocks I. DOI: 10.1107/S1600536811008737/kj2172Isup2.hkl

Additional supplementary materials:  crystallographic information; 3D view; checkCIF report

## Figures and Tables

**Table 1:** Selected bond lengths (Å)

K1—O1	2.7371 (10)
K1—O2^i^	2.8193 (10)
K1—O11^i^	2.8507 (11)
Co1—O1	2.0943 (10)
Co1—O2^ii^	2.1015 (10)
Co1—O11^iii^	2.1026 (9)

Symmetry codes: (i) 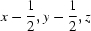; (ii) 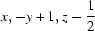; (iii) 
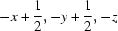
.

**Fig. 1.**
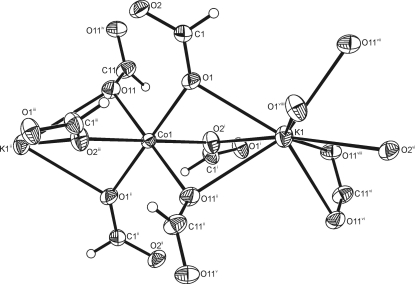
: Crystal structure of the title compound with labelling and displacement ellipsoids drawn at the 50 % probability level. Symmetry codes: i = -x+1/2, y-1/2, -z+1/2; ii = -x+1/2, -y+1/2, -z; iii = +x, -y+1, +z-1/2; iv = -x+1, +y, -z+1/2; v = +x-1/2, -y+1/2, +z-1/2.

**Fig. 2.**
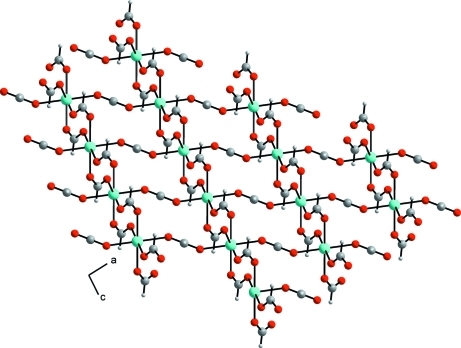
Crystal structure of the title compound with view along the crystallographic b axis. The K+ cations are omitted for clarity.

**Fig. 3.**
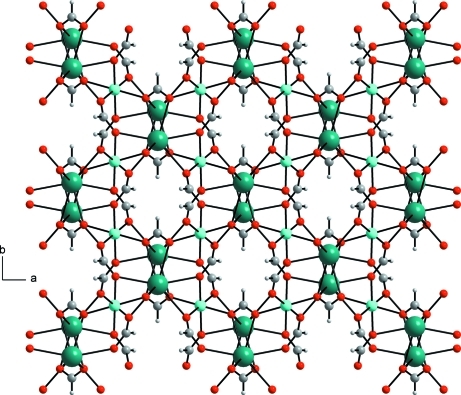
Crystal structure of the title compound with view along the crystallographic c axis.

## supplementary crystallographic information

### Comment

In our current investigation on the synthesis, structures and properties of new coordination polymers based on paramagnetic transition metal, small-sized anions and N-donor ligands, we have shown that thermal decomposition reactions are an elegante route for the discovery and preparation of new ligand-deficient coordination polymers (Boeckmann *et al.*,  2010; Wriedt & Näther, 2010; Wriedt *et al.*,  2009). Within this project we tried to prepare new ligand-rich precursor compounds based on cobalt formate and pyrazine as coligand. However, reaction of cobalt(II) chloride, potassium formate and pyrazine in acteonitrile unexpectedly resulted in single crystals of the title compound.

In the crystal structure of the title compound, each cobalt(II) cation is coordinated by six bridging formato anions with Co—OCHO distances between 2.0943 (10) Å and 2.1026 (9) Å. The CoO_6_ octahedron is slightly distorted with angles ranging from 82.66 (4) ° to 97.34 (4) ° and 180° (Fig. 1 and Tab. 1). The K^+^ cations are coordinated by eight oxygen atoms belonging to seven formato anions within irregular polyhedra. The K—O distances ranges from 2.7371 (10) Å to 2.8507 (11) Å and the O—K—O angles are between 59.81 (3) ° and 147.11 (3) °. The cobalt cations are connected via *µ*-1,3 bridging formato anions into a three dimensional coordination network (Fig. 2). Within this networks cavities are formed in which the K^+^ cations are embedded (Fig 3). The shortest Co···Co distances amount to 5.6487 (3) Å and the shortest K···K distances are 3.9067 (4) Å).

According to a search in the CCDC database (ConQuest Ver.1.12.2010) (Allen, 2002) mixed cobalt and potassium formates are unkown but bimetallic compounds based on potassium formate are known with different metals (Antsyshkina *et al.*, 1983 and Leontiev *et al.*, 1988.

### Experimental

Potassium formate (KCHOO) and pyrazine were obtained from Alfa Aesar and cobalt(II) chloride was obtained from Acros Organics. All chemicals were used without further purification. 0.25 mmol (32.5 mg) CoCl_2_, 0.5 mmol (42.1 mg) KCHOO and 0.5 mmol (40 mg) pyrazine were reacted with 1 ml acetonitrile in a closed test-tube at 120°C for three days. On cooling block-shaped single crystals of the title compound were obtained in a mixture with an unknown phase. It must be noted, that the reaction under similar conditions without pyrazine does not lead to the formation of the title compound.

### Refinement

The H atoms were positioned with idealized geometry and were refined isotropic with *U*_iso_(H) = 1.2*U*_eq_(C) and C—H distances of 0.93 Å using a riding model.

### Crystal data

**Table:**

[CoK(CHO_2_)_3_]	*F*(000) = 460
*M**_r_* = 233.08	*D*_x_ = 2.353 Mg m^−^^3^
Monoclinic, *C*2/*c*	Mo *K*α radiation, λ = 0.71073 Å
Hall symbol: -C 2yc	Cell parameters from 6120 reflections
*a* = 10.7244 (8) Å	θ = 3.0–29.2°
*b* = 8.9653 (6) Å	µ = 3.22 mm^−^^1^
*c* = 6.8742 (5) Å	*T* = 293 K
β = 95.539 (6)°	Block, light blue
*V* = 657.85 (8) Å^3^	0.16 × 0.09 × 0.06 mm
*Z* = 4	

### Data collection

**Table:**

Stoe IPDS-2 diffractometer	892 independent reflections
Radiation source: fine-focus sealed tube	853 reflections with *I* > 2σ(*I*)
graphite	*R*_int_ = 0.031
ω scans	θ_max_ = 29.2°, θ_min_ = 3.0°
Absorption correction: numerical (*X-SHAPE* and *X-RED32*; Stoe & Cie, 2008)	*h* = −14→14
*T*_min_ = 0.711, *T*_max_ = 0.817	*k* = −12→12
6120 measured reflections	*l* = −9→9

### Refinement

**Table:**

Refinement on *F*^2^	Secondary atom site location: difference Fourier map
Least-squares matrix: full	Hydrogen site location: inferred from neighbouring sites
*R*[*F*^2^ > 2σ(*F*^2^)] = 0.020	H-atom parameters constrained
*wR*(*F*^2^) = 0.046	*w* = 1/[σ^2^(*F*_o_^2^) + (0.0276*P*)^2^ + 0.1263*P*] where *P* = (*F*_o_^2^ + 2*F*_c_^2^)/3
*S* = 1.15	(Δ/σ)_max_ < 0.001
892 reflections	Δρ_max_ = 0.25 e Å^−^^3^
54 parameters	Δρ_min_ = −0.57 e Å^−^^3^
0 restraints	Extinction correction: *SHELXL97* (Sheldrick, 2008), Fc^*^=kFc[1+0.001xFc^2^λ^3^/sin(2θ)]^-1/4^
Primary atom site location: structure-invariant direct methods	Extinction coefficient: 0.0126 (12)

### Special details

**Table:**

Geometry. All esds (except the esd in the dihedral angle between two l.s. planes) are estimated using the full covariance matrix. The cell esds are taken into account individually in the estimation of esds in distances, angles and torsion angles; correlations between esds in cell parameters are only used when they are defined by crystal symmetry. An approximate (isotropic) treatment of cell esds is used for estimating esds involving l.s. planes.
Refinement. Refinement of F^2^ against ALL reflections. The weighted R-factor wR and goodness of fit S are based on F^2^, conventional R-factors R are based on F, with F set to zero for negative F^2^. The threshold expression of F^2^ > 2sigma(F^2^) is used only for calculating R-factors(gt) etc. and is not relevant to the choice of reflections for refinement. R-factors based on F^2^ are statistically about twice as large as those based on F, and R- factors based on ALL data will be even larger.

### Fractional atomic coordinates and isotropic or equivalent isotropic displacement parameters (Å^2^)

**Table:**

	*x*	*y*	*z*	*U*_iso_*/*U*_eq_	
K1	0.0000	0.10357 (5)	0.2500	0.02582 (12)	
Co1	0.2500	0.2500	0.0000	0.01561 (10)	
O1	0.16793 (10)	0.33291 (11)	0.24238 (15)	0.0274 (2)	
O2	0.25445 (9)	0.54804 (11)	0.34563 (15)	0.0271 (2)	
C1	0.18397 (13)	0.44031 (15)	0.3568 (2)	0.0244 (3)	
H1	0.1371	0.4392	0.4638	0.029*	
O11	0.43048 (9)	0.31029 (12)	0.12114 (14)	0.0260 (2)	
C11	0.5000	0.2478 (2)	0.2500	0.0271 (4)	
H11	0.5000	0.1441	0.2500	0.033*	

### Atomic displacement parameters (Å^2^)

**Table:**

	*U*^11^	*U*^22^	*U*^33^	*U*^12^	*U*^13^	*U*^23^
K1	0.0278 (2)	0.01932 (19)	0.0318 (2)	0.000	0.01004 (16)	0.000
Co1	0.01611 (14)	0.01374 (14)	0.01648 (14)	−0.00041 (8)	−0.00102 (8)	0.00122 (8)
O1	0.0332 (5)	0.0223 (5)	0.0277 (5)	−0.0087 (4)	0.0091 (4)	−0.0093 (4)
O2	0.0300 (5)	0.0204 (4)	0.0318 (5)	−0.0063 (4)	0.0076 (4)	−0.0089 (4)
C1	0.0312 (6)	0.0213 (6)	0.0215 (6)	−0.0042 (5)	0.0069 (5)	−0.0037 (5)
O11	0.0215 (4)	0.0303 (5)	0.0245 (5)	−0.0036 (4)	−0.0063 (4)	0.0033 (4)
C11	0.0270 (9)	0.0217 (9)	0.0310 (10)	0.000	−0.0057 (8)	0.000

### Geometric parameters (Å, °)

**Table:**

K1—O1	2.7371 (10)	Co1—O11^iv^	2.1026 (9)
K1—O2^i^	2.8193 (10)	O1—C1	1.2448 (17)
K1—O11^ii^	2.8335 (10)	O2—C1	1.2335 (17)
K1—O11^i^	2.8507 (11)	C1—H1	0.9300
K1—C11^i^	3.189 (2)	O11—C11	1.2356 (13)
Co1—O1	2.0943 (10)	C11—H11	0.9300
Co1—O2^iii^	2.1015 (10)		
			
O1—K1—O1^v^	82.62 (5)	O1—Co1—O11^iv^	87.99 (4)
O1—K1—O2^i^	140.19 (3)	O1^iv^—Co1—O11^iv^	92.01 (4)
O1^v^—K1—O2^i^	59.81 (3)	O2^iii^—Co1—O11^iv^	94.96 (4)
O2^i^—K1—O2^vi^	159.66 (4)	O2^vi^—Co1—O11^iv^	85.04 (4)
O1—K1—O11^ii^	92.48 (3)	O11^iv^—Co1—O11	180.00 (6)
O1^v^—K1—O11^ii^	63.08 (3)	C1—O1—Co1	137.25 (9)
O2^i^—K1—O11^ii^	60.35 (3)	C1—O1—K1	128.31 (9)
O2^vi^—K1—O11^ii^	126.22 (3)	Co1—O1—K1	94.38 (3)
O11^ii^—K1—O11^iv^	148.37 (5)	C1—O2—Co1^vii^	126.81 (9)
O1—K1—O11^i^	147.11 (3)	C1—O2—K1^viii^	138.13 (9)
O1^v^—K1—O11^i^	123.09 (3)	Co1^vii^—O2—K1^viii^	91.88 (3)
O2^i^—K1—O11^i^	71.79 (3)	O2—C1—O1	127.90 (13)
O2^vi^—K1—O11^i^	89.25 (3)	O2—C1—H1	116.0
O11^ii^—K1—O11^i^	116.58 (3)	O1—C1—H1	116.0
O11^iv^—K1—O11^i^	93.17 (3)	C11—O11—Co1	129.50 (9)
O11^i^—K1—O11^vi^	45.45 (4)	C11—O11—K1^iv^	125.17 (6)
O1—K1—C11^i^	138.69 (2)	Co1—O11—K1^iv^	91.47 (3)
O2^i^—K1—C11^i^	79.83 (2)	C11—O11—K1^viii^	94.23 (9)
O11^ii^—K1—C11^i^	105.81 (2)	Co1—O11—K1^viii^	124.29 (4)
O11^i^—K1—C11^i^	22.727 (19)	K1^iv^—O11—K1^viii^	86.83 (3)
O1—Co1—O1^iv^	180.0	O11^ix^—C11—O11	126.09 (18)
O1—Co1—O2^iii^	97.34 (4)	O11^ix^—C11—K1^viii^	63.04 (9)
O1^iv^—Co1—O2^iii^	82.66 (4)	O11—C11—H11	117.0
O2^iii^—Co1—O2^vi^	180.00 (3)	K1^viii^—C11—H11	180.0

Symmetry codes: (i) *x*−1/2, *y*−1/2, *z*; (ii) *x*−1/2, −*y*+1/2, *z*+1/2; (iii) *x*, −*y*+1, *z*−1/2; (iv) −*x*+1/2, −*y*+1/2, −*z*; (v) −*x*, *y*, −*z*+1/2; (vi) −*x*+1/2, *y*−1/2, −*z*+1/2; (vii) −*x*+1/2, *y*+1/2, −*z*+1/2; (viii) *x*+1/2, *y*+1/2, *z*; (ix) −*x*+1, *y*, −*z*+1/2.
